# Myelination of Callosal Axons Is Hampered by Early and Late Forelimb Amputation in Rats

**DOI:** 10.1093/texcom/tgaa090

**Published:** 2020-11-27

**Authors:** Rodrigo Vianna-Barbosa, Carlomagno P Bahia, Alexandre Sanabio, Gabriella P A de Freitas, Rodrigo F Madeiro da Costa, Patricia P Garcez, Kildare Miranda, Roberto Lent, Fernanda Tovar-Moll

**Affiliations:** 1 Post-Graduate Program in Morphological Sciences, Institute of Biomedical Sciences, Federal University of Rio de Janeiro, Rio de Janeiro CEP 21941-902, Brazil; 2 National Center of Structural Biology and Bioimaging, Federal University of Rio de Janeiro, Rio de Janeiro CEP 21941-902, Brazil; 3 Institute of Health Sciences, Federal University of Pará, Pará CEP 66035-160, Brazil; 4 D’Or Institute of Research and Education (IDOR), Rio de Janeiro, CEP 22281-100, Brazil; 5 Institute of Biophysics, Federal University of Rio de Janeiro, Rio de Janeiro CEP 21941-902, Brazil

**Keywords:** amputation, corpus callosum, morphological plasticity, myelination, somatosensory cortex

## Abstract

Deafferentation is an important determinant of plastic changes in the CNS, which consists of a loss of inputs from the body periphery or from the CNS itself. Although cortical reorganization has been well documented, white matter plasticity was less explored. Our goal was to investigate microstructural interhemispheric connectivity changes in early and late amputated rats. For that purpose, we employed diffusion-weighted magnetic resonance imaging, as well as Western blotting, immunohistochemistry, and electron microscopy of sections of the white matter tracts to analyze the microstructural changes in the corticospinal tract and in the corpus callosum (CC) sector that contains somatosensory fibers integrating cortical areas representing the forelimbs and compare differences in rats undergoing forelimb amputation as neonates, with those amputated as adults. Results showed that early amputation induced decreased fractional anisotropy values and reduction of total myelin amount in the cerebral peduncle contralateral to the amputation. Both early and late forelimb amputations induced decreased myelination of callosal fibers. While early amputation affected myelination of thinner axons, late amputation disrupted axons of all calibers. Since the CC provides a modulation of inhibition and excitation between the hemispheres, we suggest that the demyelination observed among callosal fibers may misbalance this modulation.

## Introduction

Neuroplasticity is the ability of the central nervous system (CNS) to reorganize and adapt in response to environmental and physiological changes (Pascual-Leone et al. 2005; Tovar-Moll and Lent 2017). These changes can occur at different levels of complexity, and brains at earlier stages of development are considered to be more plastic than mature ones (Bowlus et al. 2003; Staudt et al. 2004; Ismail et al. 2017). CNS plasticity, therefore, tends to manifest differently along time due to modulation of molecular and cellular mechanisms which influence brain cellularity, synaptogenesis, and formation of long-distance connectivity. Age, therefore, determine different “time windows” known as critical periods, specific to each neural aspect, during which the CNS shows higher susceptibility to change (Ismail et al. 2017). However, adult mammals also present a certain degree of plastic potential, albeit subtler, mainly perceived at synaptic and short-distance axonal connectivity levels (Cornelissen et al. 2003; Bavelier et al. 2010; Chen et al. 2011; Ueno et al. 2019).

One of the major causes of neuroplastic phenomena is deafferentation, a loss of nervous inputs originating from the peripheral organs or from the CNS itself (Humanes-Valera et al. 2017; Ramalho et al. 2019; Vierck 2019) that may lead to different kinds of brain changes. In mammals, these inputs from the body periphery first reach the neocortex at the primary somatosensory area (S1), which contains a representation map of the whole body (Zembrzycki et al. 2013). Deafferentation of S1, thus, leads not only to map alterations (Bowlus et al. 2003; Wu et al. 2016) but also to changes in the dynamic expression of neuromediators therein (Gierdalski et al. 1999; He et al. 2004), and structural changes in related white matter bundles such as the corpus callosum (CC) and the pyramidal tract (Lindenberg et al. 2012; Simões et al. 2012).

The CC is the largest white matter bundle of placental mammals, composed by about 200 million fibers in humans (Rapoport 1990; Aboitiz et al. 1992; Suárez et al. 2018), that connect homotopic and heterotopic areas of both cerebral hemispheres and are essential for coordinated transfer of information between the sensory and motor cortices (Donahoo and Richards 2009; Suárez et al. 2014b).

The pyramidal tract is part of the corticofugal pathways and is composed of corticorubrospinal, corticoreticulospinal, and corticospinal fibers. It originates primarily from the premotor and primary motor cortices, which send axons through the internal capsule and cerebral peduncle, with an important role in motor control (Gribnau et al. 1986; Matsuyama et al. 2004; Lindenberg et al. 2012).

A previous study by our group, employing diffusion tensor imaging (DTI), has shown a decreased fractional anisotropy (FA) in the CC of patients who had suffered lower limb amputation. This decrement was specifically localized at the sector of the CC that contains axons connecting S1 of both hemispheres (Simões et al. 2012). FA value within the white matter is usually related to its degree of myelination, so that the higher the FA, the more myelinated the bundle is supposed to be (Yuan et al. 2010; Choi et al. 2015; Leong et al. 2015). That means that after different kinds of injuries, including deafferentation, white matter bundles might undergo structural changes related to their myelination degree, which could be detected by FA analysis.

On the other hand, it was also shown that myelination degree is related with neuronal activity (Stedehouder et al. 2018; Choi et al. 2019). An increment of neuronal electrical activity in the mouse premotor cortex causes a mitogenic response of oligodendrocyte precursor cells (OPCs) and increases myelination of the subcortical white matter, followed by motor function improvement (Gibson et al. 2014). Also, an in vitro study has demonstrated that neurons co-cultivated with oligodendrocytes, when exposed to several sessions of patterned electrical stimulation, showed a frequency-dependent increase of axonal myelination, which can be suppressed by inhibiting the cyclic adenosine monophosphate (cAMP) pathway. Therefore, electrical activity promotes myelination modulated by intrinsic neural signaling (Malone et al. 2013). Moreover, experiments in rats have demonstrated a decreased functional activity between S1 of both hemispheres caused by limb deafferentation (Pawela et al. 2010), which places the CC and the pyramidal tract as possible targets to myelination changes caused by amputation.

Based on previous findings of FA decrease in the CC of human amputees (Simões et al. 2012), and a recently published study showing changes of callosal axons at the opposite hemisphere (Bahia et al. 2018), the main goal of this work was to investigate the microstructural white matter changes induced by experimental deafferentation through forelimb amputation in rats, performed both in early (neonate) and late life (adult) animals. We confirmed that, after amputation, crossed corticocortical and corticofugal axons at the corpus callosum and the cerebral peduncle, respectively, presented FA decrease and demyelination, supporting the hypothesis raised by imaging studies in humans, and strengthening the interpretation that cortical somatotopic maps may be impacted by deficient conduction of efferent information to the opposite hemisphere and to subcortical targets.

## Materials and Methods

### Animals

All experiments were authorized by the Ethics Committee on the Use of Animals of our university and complied with international norms as detailed by the “Guide for the Care and Use of Laboratory Animals” (NIH publication, No. 86-23, revised 1985). A total of 37 adult male rats were used in this study, divided into 3 different groups (Table 1): Topography (T), Early amputation (E), and Late amputation (L). Group T was intended to locate the precise callosal sector that contains axons from the forelimb representation in the cerebral cortex, by using an axonal tracer injected into the cortex, under electrophysiological guidance. Groups E and L were composed of animals that underwent a right forelimb amputation at the first postnatal (P) day (group E) or at P90 (group L). The control groups were composed of non-operated animals paired by sex and age to groups E or L. After the amputation, all animals survived 90 days before being euthanized by a lethal dose of 100-mg/kg ketamine and 5-mg/kg xylazine. The brains of groups E and L were dissected and subjected to magnetic resonance protocols, as well as immunohistochemistry and electron microscopy analyses of the white matter. A subset of brains (3 from controls and 3 from amputees) from group E was dissected and subjected to Western blotting of two different regions: 1) the sector of corpus callosum connecting the forepaw representations in S1 of both hemispheres and 2) the cerebral peduncle at pontine level.

**Table 1:** Animal groups and corresponding techniques employed (# = number of animals).

Groups	Subgroups	Electro-physiology	Axonal tracer	Diffusion imaging	Immuno-histochemistry	Western blotting	Electron microscopy	Total number of animals
T (#)	Experimental	✓ (3)	✓ (3)					3
E (#)	Experimental			✓ (7)	✓ (7)	✓ (3)	✓ (7)	10
	Controls			✓ (7)	✓ (7)	✓ (3)	✓ (7)	10
L (#)	Experimental			✓ (7)	✓ (7)		✓ (7)	7
	Controls			✓ (7)	✓ (7)		✓ (7)	7

### Electrophysiological Recording and BDA Injection (Group T)

Standard multiunit recording and receptive field mapping techniques were used to assess the forepaw representation in S1, as described previously (Bahia et al. 2018). Briefly, adult male rats (>P60) were anesthetized with a combination of 100-mg/kg ketamine and 20-mg/kg xylazine injected intraperitoneally (IP), then placed into a stereotaxic head holder. Body temperature was maintained by a thermal blanket within the range of 36–38°C during the whole surgery. A midline incision was made in the scalp to expose the skull, then a small sector of the cranium overlying the presumptive somatosensory cortex was removed, and finally the dura-mater was opened to allow access to the cortex. Multiunit responses were recorded with varnish-coated tungsten microelectrodes and cutaneous response fields were mapped with tactile stimuli delivered at the forepaw with a brush.

Once the representation of the forepaw was found, the microelectrode was replaced by a glass micropipette filled with 10% Biotinylated Dextran Amine 10 KDa (BDA, Molecular Probes) diluted in saline phosphate buffer. One microliter of BDA solution was injected into the cortex at about 500 μm depth using a microsyringe (Hamilton, 1 μL) filled with oil. After injection, the micropipette was removed, the skull was closed with methacrylate and the skin was sutured. After a 15-day survival, the animals were euthanized and the tissue preparation procedures were performed as described below.

### Forelimb Amputations (Groups E and L)

Neonatal limb amputation was performed as previously described (Lane et al. 1995; Bahia et al. 2018). Pups (<12 h old) were anesthetized by hypothermia until becoming immobile and insensitive to mild painful stimuli. The right forelimb was then amputated close to the shoulder using iridectomy scissors, and the brachial artery was sealed by electrocauterization. The stump was infiltrated with 0.2 mL of a local anesthetic (0.7% Bupivacaine, Nortec Quimica), and the skin was sealed with cyanoacrylate. After surgery, the animals were rewarmed and returned to their mothers. These animals were euthanized and studied 90 days after amputation.

Adult amputations were performed on male rats at P90. The animals were anesthetized with 100-mg/kg Ketamine Hydrochloride (Ketalar, Parker), and 20-mg/kg xylazine (Rompun, Bayer) injected intramuscularly (IM), after which a skin incision was made at the forearm and the brachial nerve was cut distal to the brachial plexus. The humerus was then exposed and transected. The adjacent muscles were freed and sutured together over the humeral stump, then the region was infiltrated with 0.5% bupivacaine and the skin was sutured. These animals were euthanized and studied 90 days after amputation.

### Tissue Preparation for Histology (All Groups)

After the survival period, animals were administered a lethal dose of ketamine and xylazine and perfused transcardially with phosphate buffer (PB) 0.1 M (pH 7.4) followed by a solution of 4% formaldehyde in phosphate buffer (pH 7.4). Animals from group T had their brains removed and sliced into 100-μm-thick sections (2 brains sliced parasagittally; 1 sliced coronally) for BDA histochemistry. Animals from groups E and L had their heads removed after perfusion and immersed into a falcon tube with phosphate buffer 0.1 M (pH 7.4) for diffusion imaging. Their brains were then removed and divided into 2 hemispheres, the right ones sliced into 100-μm-thick coronal sections for immunohistochemistry against Myelin Basic Protein (MBP), and the left hemispheres fixed with a solution containing 2.5% glutaraldehyde and 4% formaldehyde in phosphate buffer (pH 7.4) for 7 days at 4 °C, for electron microscopy.

### BDA Histochemistry (Group T)

Sections were washed three times in PB (20 min each) and once in a solution of 3% Triton X-100 in PB, before being incubated overnight, free-floating in the avidin/biotin/peroxidase complex (ABC 1:200; Vector Laboratories) at room temperature, under constant agitation. Peroxidase labeling was shown using the Diaminobenzidine reaction intensified with nickel ammonium sulfate (Hellwig 2000). Finally, sections were dehydrated in rising ethanol concentrations and cleared in xylene. Coverslips were gently placed over the samples mounted with Entellan (Merck).

### ROI Delimitation (Group T)

**Figure 1:**
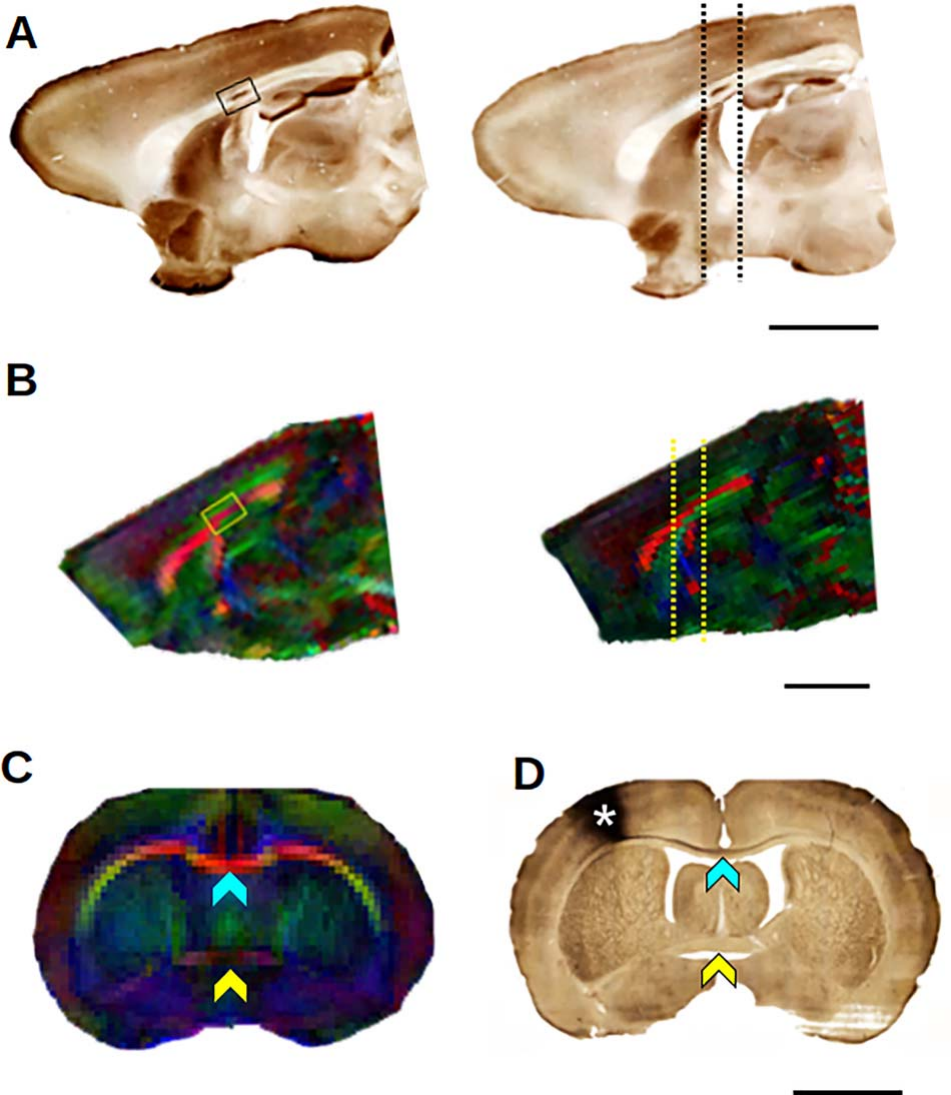
ROI delimitation of the somatosensory sector of the corpus callosum. (*A*) Parasagittal sections of the brains of two normal rats that underwent BDA injection at the forelimb representation of primary somatosensory cortex. The black rectangle shows the BDA-stained callosal sector. The black dotted lines on the right depict the coronal levels of slices as shown in D. (*B*) Sagittal views of FA-colored maps of two other control rats. Yellow rectangle shows the region of interest equivalent to those shown in (*A*). The yellow dotted lines indicate the level range of the coronal plane shown in (*C*). (*C*) Coronal view of FA colored map of a control rat. The blue arrowhead shows the corpus callosum, and the yellow one shows the anterior commissure. (*D*) Coronal section of the brain of a normal rat that underwent a BDA injection at the forelimb representation of primary somatosensory cortex at the right side. The blue arrowhead shows BDA stained fibers along the corpus callosum. The yellow arrowhead shows the anterior commissure, and the asterisk indicates the BDA injection site. Scale bars = 250 μm.

The BDA staining of the sagittal sections of group T animals (Fig. 1*A*) was used to determine the sector of the corpus callosum that contains axons connecting the forelimb representations between both hemispheres (Fig. 1*A*). The position of the stained fibers within the CC encompassed about 6.5% average area, consistently at about half the sagittal length of the CC. The coronal sections (Fig. 1*D*) revealed that the majority of stained callosal axons were located consistently at the same coronal level as that of the anterior commissure. This observation agreed with a previous study on rat interhemispheric connectivity of S1 forelimb representation after BDA injections (Zakiewicz et al. 2011). It was also confirmed by 3D axonal reconstructions in a previous work by our group (Bahia et al. 2018). The same anatomical markers and the relationship with the anterior commissure were used to define the manual drawing of the region of interest (ROI) in the sagittal diffusion images (Fig. 1*B*, yellow square) and the coronal section slices for the immunohistochemistry reaction for MBP staining.

### MBP Immunohistochemistry (Groups E and L)

The sections obtained from the right hemispheres of animals from groups E and L were pre-incubated in 10% normal goat serum in Tris-Buffer (Vector Laboratories) for 1 h. This step was followed by overnight incubation in mouse primary anti-MBP antibody (1:250, Novocastra), in a humidified chamber at 4 °C. Sections were then washed with phosphate buffer 0.1 M (pH 7.4) and incubated in the secondary anti-mouse antibody (1:400, Invitrogen, EUA) for 2 h at room temperature, followed by DAPI staining for 20 min, then washed with phosphate buffer 0.1 M (pH 7.4) and finally mounted onto histological glass slides with Fluoromount (Invitrogen) to decrease photobleaching. Images of selected sections were acquired with a confocal microscope (Leica TCS SP5), the fluorescence intensity was measured, and Student’s *t*-test was used to compare sections with and without the primary antibody in experimental and controls animals.

### Western Blotting (Group E)

Protein extracts from the regions of interest in the CC and in the cerebral peduncle were obtained by lysis with RIPA buffer (Sigma). The protein concentration was measured using the BCA protein assay kit (Thermo Scientific). Twenty micrograms of protein for each condition was applied on a 12% SDS polyacrylamide gel. After being separated by electrophoresis, proteins were transferred for 17 min to a PVDF membrane (Millipore) using the Transblot system (Bio-Rad). Then, the membrane was incubated with blocking solution (5% milk diluted in Tris-buffered saline added with 0.1% Tween 20) for 90 min. The membrane was incubated with primary antibodies anti alpha-tubulin (1:2000, Sigma) and anti-MBP (1:1000, Novocastra) diluted in 1% Milk overnight, followed by 1-h incubation with the secondary antibodies IRDye 680CW goat anti-mouse (LI-COR) or IRDye 800CW goat anti-rabbit antibody (LI-COR). The effect size was measured by calculating the Cohen’s *d* value.

### Transmission Electron Microscopy (Groups E and L)

The right hemispheres of animals from groups E and L were sliced coronally with a vibratome into 20-μm-thick sections until the first section including the anterior commissure at the midline. At this level, a thicker section of 500 μm was obtained, and the corpus callosum within it was dissected to expose its sagittal surface and fixed with a solution containing 2.5% glutaraldehyde and 4% formaldehyde in PB pH 7.4 for 24 h at 4 °C. The callosal blocks were then post-fixed in 1% osmium tetroxide in 0.1 M PB for 40 min. After dehydration in ascending acetone series, the tissue blocks were embedded in epoxy-resin and 70-nm-thick ultrathin sections were obtained, then reacted with uranyl acetate (1%) for 30 min, and finally examined at a Jeol 1200EX or a Tecnai Spirit electron microscopes operating at 80 kV.

Fifteen fields of each 3 experimental and control animals of both groups were used to randomly select 150 axons of each animal. We then measured the inner and outer diameters of the myelin sheaths and calculated their *g*-ratio (inner diameter/outer diameter of myelinated fiber) values, and a Student’s *t* test was used to compare them.

### Diffusion Weighted Imaging Acquisition and Post-Processing (Groups E and L)

Animals from groups E and L were submitted to an ex vivo imaging protocol, including diffusion-weighed acquisition in a 7-T magnetic resonance scanner (Varian). Data were acquired using spin echo sequence (TR = 4 s, TE = 24 ms, matrix 128 × 128, FOV = 40 × 40 mm, *b* value = 1000 cm^3^/s) in six directions and one data set without diffusion weighting. Sixteen averages were acquired, and the total scanning time was 15 h 55 min.

For the analysis of diffusion-weighted images, a binary mask for brain extraction was made manually in *flsview*, then the diffusion tensor was determined with the dtifit software provided by FSL (Smith et al. 2004), and fractional anisotropy (FA) maps were calculated. After that, the ROI was manually drawn within the CC (for site specification see the section above) in the 2 most medial slices with *fslview*, FA values were extracted, and Student’s *t*-test was used to compare experimental and controls animals. In addition, to assess the global differences in white matter fiber tracts between the experimental and controls animals, whole-brain voxel-wise statistical analyses of FA data were conducted using tract-based spatial statistics (TBSS) (Smith et al. 2006).

Each FA image was first registered to every other image, and the one requiring minimum transformation was selected as best registration target. The target image was used as a template within which final transformations were performed. All FA data were aligned into a common space using the nonlinear registration tool FNIRT. Following registration, the mean FA image was created and thinned to represent the mean FA skeleton, and thresholded at level 0.2. Each aligned FA image was projected onto this skeleton, and the resulting data fed into voxel-wise cross-subject statistics. The data in the TBSS statistics was built over 10 000 permutations, and the results are shown as corrected for multiple comparisons voxel-wise *P* < 0.05.

## Results

We hypothesized that limb amputation could cause structural plasticity in major white matter tracts related to somatosensory limb brain circuitry, such as the corticospinal tract and the CC. To test this hypothesis and quantify those possible changes, we performed a multi-modal investigation approach, including ex vivo magnetic resonance imaging, histological and biochemical analyses, as well ultrastructural morphological quantification.

### Limb Amputation Induces Fractional Anisotropy White-Matter Changes

**Figure 2:**
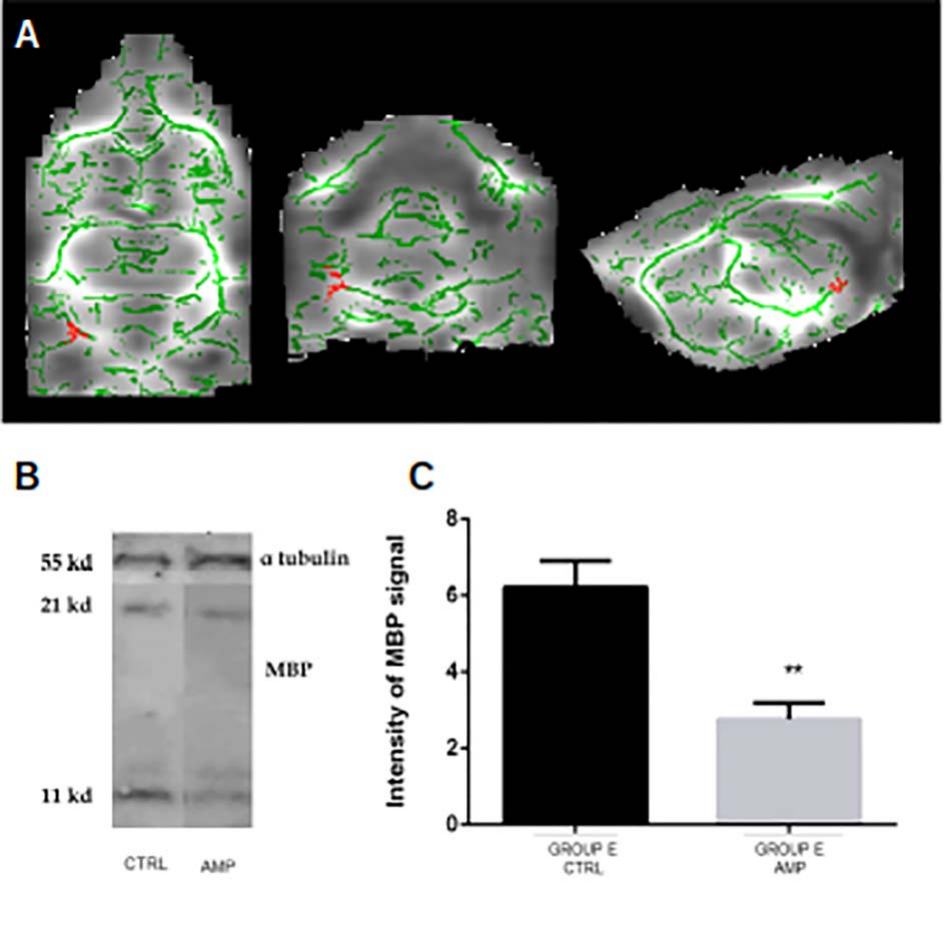
Evidence for demyelination of the cerebral peduncle in early amputees as compared with controls. (*A*) DTI voxel-wise analysis of whole brain white matter. Horizontal (left), coronal (center), and sagittal (right) view. Green: white matter bundles (FA value threshold 0.2) red: reduced FA value of cerebral peduncle in early-amputated animals compared with controls. Whole-brain TBSS, *P* < 0.05, corrected. (*B*) Western blot of MBP showing one band at 18.5 kDa and α-tubulin as loading control at 55 kDa. (*C*) Western blots fluorescence quantification. Asterisks show a significant difference between amputated and adult animals, *P* < 0.05. MBP = myelin basic protein; CTRL = controls; AMP = amputees; E = early; L = late.

To investigate possible microstructural changes detected by quantitative diffusion images, we performed a whole-brain voxel-wise statistical analysis (TBSS) of the FA maps among the three groups (controls, early, and late amputees). Our results showed significantly lower FA values in the cerebral peduncle contralateral to the missing limb, in early amputated rats when compared with control animals (*P* < 0.05, corrected) (Fig. 2*A*).

We then moved to investigate specific diffusion metrics changes in the sector of the CC connecting somatosensory forelimb representation cortices. We approached this issue by performing a ROI-based FA analysis. The mean FA values of the CC found in amputated animals were smaller than those of the controls within the sector occupied by somatosensory connectivity. However, the difference did not reach significance, neither between the FA of early amputated animals (0.69 SD: 0.047) and their respective controls (0.74 SD: 0.067) (*P* = 0.079), nor between adult amputated rats (0.66 SD: 0.048) and their controls (0.7 SD: 0.073) (*P* = 0.078). This borderline result could be interpreted as due to insufficient power of this MRI experiment. To address this issue, we continued to investigate possible white matter track changes induced by amputation using more sensitive techniques as described below.

### Limb Amputation Impacts Myelination of the CC and Cerebral Peduncle

**Figure 3:**
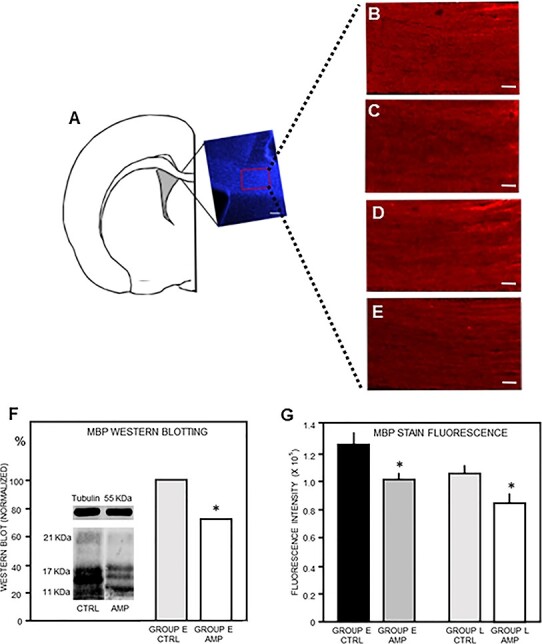
Evidence for demyelination of the callosal sector of somatosensory connections in amputees as compared with controls. (*A*) Schematic drawing of a coronal section, with the inset showing the corresponding section stained with DAPI. The red rectangle shows the region of interest where fluorescence intensity was measured with higher magnification as shown in (*B*–*E*). (*B–E*) MBP immunostaining in control (*B*) and amputated animals (*C*) of group E, and control (*D*) and amputated animals of group L (*E*). Scale bar = 100 μm. (*F*) Western blots fluorescence measurement showing the reduction of MBP levels in the amputated compared with the control. (*G*) MBP staining fluorescence quantification. ^*^P < 0.05. MBP = myelin basic protein; CTRL = controls; AMP = amputees; E = early; L = late.

Based in our hypothesis and in the results obtained from our previous imaging analyses, in this second approach we aimed to investigate possible changes of white matter myelin amount in amputees. Western blot analysis was performed in protein extracts from the cerebral peduncle (Fig. 2*B*,*C*) and from the CC sector that connects S1 forepaw representation across both hemispheres to quantify myelin basic protein (MBP) (Fig. 3*A*).

Confirming the relationship between FA metrics values and myelination, the amount of MBP in the cerebral peduncle (contralateral to the amputation) assessed by western blotting showed a significant decrease in early-amputated animals, as compared with controls (Fig. 2*B*,*C*). The same was found for the CC, which also showed a significant decrease in MBP amount in early-amputated animals, as compared with controls (Fig. 3*B*). In a complementary analysis employing anti-MBP immunohistochemistry of the same CC sector (Fig. 3*A*), we found a significant decrease of MBP staining fluorescence intensity (Fig. 3*C*) in both early (1.1 × 10^6^; SD: 5.7 × 10^4^) and late amputated (8.4 × 10^5^; SD: 7.5 × 10^4^) animals, compared with their age paired controls (1.3 × 10^6^; SD: 8.3 × 10^4^ and 1.0 × 10^6^; SD: 5.7 × 10^4^) (*P* < 0.05), respectively.

### Ultrastructural Changes of CC Fibers in Amputees

**Figure 4:**
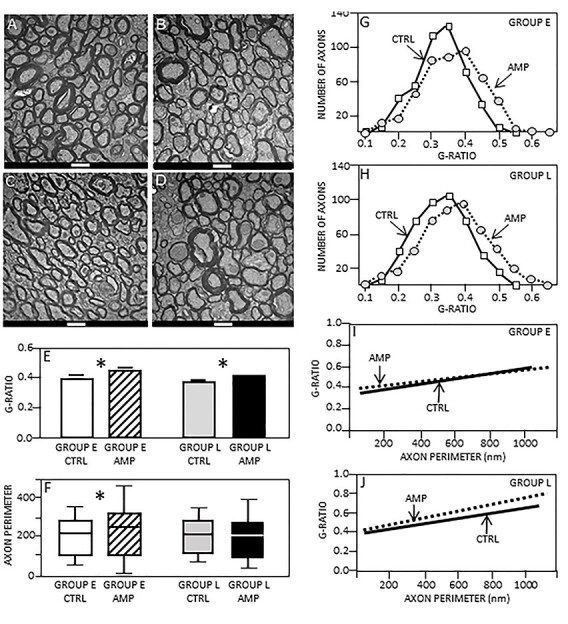
Electron microscopy (EM) analysis of the callosal sector representing the forepaw axons. (*A–D*) EM microphotographs within the forepaw callosal sector of different animals. (*A*) Control animal of group E. (*B*) Amputated animal of group E. (*C*) Control animal of group L. (*D*) Amputated animal of group L. Scale bars = 1 μm. (*E*) and (*F*), *g*-ratios and axonal perimeters, respectively, in control versus amputated animals. Asterisks show a significant difference between amputated and adult animals, *P* < 0.01. (*G*) and (*H*) Plots of the average number of axons against their *g*-ratios in early (*G*) and late (*H*) amputated animals. (*I*) and (*J*) Linear regression analysis of group E (*I*) and L (*J*). *R*^2^ value for group E controls = 0.26; for amputees = 0.21; for group L controls = 0.14; and for amputees = 0.31. No correlation between *g*-ratio and perimeter of axons was found. CTRL = controls; AMP = amputees; E = early; L = late.

In order to confirm whether the detected decrease of MBP levels was due to changes in myelination of specific axons, we performed a detailed electron microscopy analysis at the same sector of the CC (Fig. 1*A*,*B*) of the different experimental groups (Fig. 4*A*–*D*). We found an increase in the *g*-ratio value of amputated animals in both early (0.43; SD: 0.09) and late (0.41; SD: 0.08) deafferented rats, as compared with their controls (0.39; SD: 0.07 and 0.38; SD: 0.07) (Fig. 4*E*). This difference could be seen also by comparing the distribution of axons according to their *g*-ratios in early (Fig. 4*G*) and late (Fig. 4*H*) amputated animals. However, this difference in *g*-ratio could be caused either by an increase of axon caliber or by a decrease of the myelin amount per axon. Therefore, to discern between these two possibilities, we measured the axon calibers. A significant difference was found between early amputated rats (250.41; SD: 231.56) and control animals (216.61 SD: 154.95), showing larger axons in amputees (Fig. 4*F*). However, no difference was found between the axon caliber of late amputated animals (214.86; SD: 186.90) and their controls (213.28; SD: 148.53). In addition, we performed a linear regression analysis to investigate a possible relationship between axonal calibers and their *g*-ratio values, but no relation was found in any of the groups. Nevertheless, different patterns of myelin distribution among axons of different calibers appeared (Fig. 4*I*,*J*). Early amputated animals showed an increase in *g*-ratio value for smaller axons, while late amputation causes a *g*-ratio increase for axons of all calibers.

Taken together, these findings reveal a difference between structural plasticity of CC caused by early as compared with late forelimb amputation. Early deafferentation determines an increase of axon caliber and demyelination of small perimeter axons, while adult or late amputation induces no change in caliber, but a more accentuated demyelination of all axons.

## Discussion

Evidence of interhemispheric plasticity has been reported in humans under different pathological conditions, including amputation and blindness (e.g., Simões et al. 2012; Wang et al. 2013), by employing different electrophysiological and neuroimaging techniques. These approaches, however, due to low morphological resolution, fail to unravel the specific microstructural changes that take place among white matter tracts. The aim of the present work was to perform a detailed morphological investigation to clarify this issue. Using a rodent model, we were able to show that early and late forelimb amputations induce structural changes in morphology of major white matter tracts, specifically the callosal axons that interconnect bilaterally the somatosensory cortical representations of the removed and remaining limbs. This effect occurred in both early and late amputees, albeit in different ranges among axons of different calibers within the same tract. In addition, we showed that amputation impacts on the contralateral corticospinal tract in early amputees.

Wang et al. (2013) demonstrated that FA of CC splenium in blind patients decreases, and previous work of our group has also shown a similar phenomenon in the CC of patients who had suffered lower limb amputation (Simões et al. 2012). The precise mechanisms that may generate this change in humans are unknown. However, it is known that the degree of myelination may impact FA values (Yano et al. 2018), since this parameter is related to the diffusional restriction of water molecules movements (Basser et al. 1994), forced to occur mainly parallel to axonal fascicles due to the hydrophobic nature of myelin sheaths (Baumann and Pham-Dinh 2001; Schmitt et al. 2015; Inouye and Kirschner 2016; Snaidero and Simons 2017).

Using an animal model of limb amputation, we found that amputation decreased FA values in the cerebral peduncle, contralateral to the amputation in early amputees. In addition, we found a tendency for FA value decrease in the CC caused by both early and late forelimb amputations; however, these changes were not statistically significant. The lack of significance of the result of corpus callosum FA analysis can be attributed to this methodology resolution, comparatively to histological or molecular techniques, FA analysis has a lower resolution and the kind of structural alteration of corpus callosum bundle caused by limb amputation observed in this work is just not big enough to be showed by FA analysis; nevertheless, it could be demonstrated by immunohistochemistry, western blotting, and transmission electron microscopy because these methodologies have higher definition than the first one. Moreover, by using the western blotting data, we were able to compare the effect size of limb amputation on myelination of corpus callosum and cerebral peduncle, this comparison showed a higher Cohen’s *d* value of cerebral peduncle data (3,95) than corpus callosum (2,65). Taken together, these findings reinforce the conclusion that amputation causes FA decrease in the main cortical tracts related to the site of deafferentation, suggesting an impact on their axonal microstructure.

The nature of these microstructural changes was uncovered by immunohistochemistry, Western blotting, and electron microscopy, indicating a reduced amount of myelination in amputated animals. Also, we could confirm the relationship between FA value and the degree of myelination, when combining neuroimaging and Western blot analysis. These approaches showed a significant reduction in total MBP amount in another white matter structure (the cerebral peduncle) that presented a clear FA value reduction after early amputation, as assessed by whole-brain voxel-wise statistical analyses.

Myelination of the central nervous system requires the generation of functionally mature oligodendrocytes from oligodendrocyte precursor cells (OPCs) and takes place at late stages of CNS development in all mammals, being one of the last steps of neural circuitry maturation (Baumann and Pham-Dinh 2001; Snaidero and Simons 2017). However, the presence of OPCs in the CC has been reported as early as 7 days after birth, until the beginning of white matter myelination below the somatosensory cortex, which occurs after the 10th postnatal day and extends through adulthood in mice (Vincze et al. 2008).

Many studies have shown a relation between neural activity and myelination (Stedehouder et al. 2018; Choi et al. 2019). Optogenetic stimulation of the premotor cortex in awake mice leads to increase in OPC proliferation (Gibson et al. 2014). Although the entire sequence of molecular mechanisms involved in this relation is poorly known, it has been shown that increase in neuronal electrical activity is accompanied by higher intracellular cAMP concentrations and is followed by increase in axonal myelination (Malone et al. 2013). Along with this evidence, it is reasonable to expect, similarly, that a decrease in neuronal activity could cause a decrease in myelination.

Forelimb amputation leads to morphological and functional plasticity of the somatosensory cortex (Pearson et al. 1999, 2003; Wu et al. 2016). Functionally, the most evident plastic phenomenon is the appearance of a new representation of remaining body regions such as the shoulder, in addition to the normal one, extending into the deafferented forelimb representation. This was reported both immediately after deafferentation and later on, being more pronounced in the former period (Pearson et al. 1999; Humanes-Valera et al. 2013, 2017).

Expansion of the stump representation in the cortex of amputees is first seen 2 weeks after a forelimb amputation. However, 4 months after amputation there still are portions of the original forelimb representation of primary somatosensory cortex that remain unresponsive to stimuli applied to any other parts of the body periphery. These unresponsive regions are larger in rats amputated as adults than in those amputated as newborns (Pearson et al. 2003). Therefore, both early and late forelimb amputation determine a decrease in neuronal activity within the forelimb representation in S1, for at least 2 weeks, which could be caused by a decrease of neurotransmitters released at thalamocortical synaptic connections. The thalamocortical circuitry is a classic glutamatergic excitatory pathway (Li et al. 2014; Matsumoto et al. 2016). Therefore, a decrease of its activity may lead to a reduction of postsynaptic firing frequency at the cortical targets. Considering the high level intracolumnar connectivity within the forelimb representation in S1 (Lübke and Feldmeyer 2007; Wagener et al. 2016), it is expected that this reduction would change the neuronal activity pattern of all cortical layers within S1, which could lead to a reduction of myelination of white matter fibers emerging from these cortical areas, as is the case of the CC and of the cerebral peduncle.

There is an experience-dependent critical period for the correct formation of functional and morphological S1 organization in rats. This critical period extends until the second week after birth (Lendvai et al. 2000; Maravall et al. 2004). It has already been shown that S1 from one hemisphere connects homo- and heterotopically to S1 and S1/S2 border of the contralateral hemisphere via the corpus callosum (Fenlon et al. 2017). Moreover, previous works have shown that unilateral deprivation of sensory activity during the critical period disrupts callosal wiring, leading to a less functional connection between S1 and contralateral S1/S2 border (Suárez et al. 2014a). Therefore, the loss of somatosensory inputs from the periphery causes not only functional but also structural change in CC axons connecting S1 cortices. One example of the latter is the enlargement of callosal axons telodendria and the increase in number of synaptic boutons in the limb region of S1 receiving input from the deafferented counterpart in the opposite hemisphere (Bahia et al. 2018).

Besides myelin formation, fiber maintenance is also mediated by intrinsic mechanisms of the axon (Popko 2010; Schmitt et al. 2015; Snaidero and Simons 2017). Rats that had their tails attached to the top of their cage by a swivel allowing 360° rotation, but preventing a contact of the hindlimbs with the ground, showed a *g*-ratio decrease of the nerves originating therein, while an increase of stimulation leads to an increment of myelination, together with an increase of axonal caliber (Canu et al. 2009). Nevertheless, studies of CC normal myelination in mice have shown a lack of correlation between *g*-ratio and axonal caliber (West et al. 2015).

There are axons of different calibers in the CNS, and this morphological diversity reflects on their functional features, so that thicker axons exhibit higher firing frequency and conduction speed than thinner ones (Perge et al. 2012). Another factor that impacts on conduction speed is the myelination degree, as more myelinated axons display higher conduction speeds (Hartline and Colman 2007; Schmitt et al. 2015; Inouye and Kirschner 2016; Snaidero and Simons 2017). The total amount of neurofilaments is the main factor for axonal caliber determination: a loss of neurofilaments causes a decrease of axonal caliber (Hoffman et al. 1984; Uchida et al. 2016; Costa et al. 2018; Walker et al. 2019). The neurofilament amount also stimulates the interaction of oligodendrocytes and axons, which finally impacts directly on myelin formation (Demerens et al. 1995; Fressinaud and Eyer 2014).

Considering the above evidence, and the fact that forelimb amputation leads to reduced myelination of CC fibers caused by a decrease of activity of callosal neurons, it is conceivable that early and late amputations may imply different mechanisms. Thus, the former would impact preferentially on myelin formation, while the latter would influence myelin maintenance. In addition, it is well known that the CC has axons of different calibers (West et al. 2015), and with different neurofilament concentrations, so that early and late amputation could affect differently axons of different calibers.

Late amputation determines the occurrence of a greater number of unresponsive regions in S1 forelimb representation (Pearson et al. 2003). Therefore, a higher degree of demyelination caused by such kind of deafferentation would be expected. On the other hand, thinner axons tend to have a lower firing frequency than thicker ones (Perge et al. 2012), being probably more sensitive to neuronal activity decreases caused by amputation, what could interfere in their signaling to oligodendrocytes, thereby impacting on their myelination. So, early amputation could lead to a neuronal activity decrease strong enough to impact mostly the myelination of thinner axons, thicker ones being protected by the residual S1 activity.

Bundles of the motor efferent system are the main component of the corticofugal pathway (Cheney 1985; Lemon 2008). Before reaching their subcortical targets, fibers arise mainly from primary motor cortex (M1) and course through the internal capsule and cerebral peduncle (Mitchell et al. 2016).

M1 receives many connections from S1 (Aronoff et al. 2010; DeNardo et al. 2015), so it is expected that disruptions of the somatosensory cortex would impact on M1. Also, several works have shown that correct development of the efferent motor system depends on normal use of the limbs (Williams and Martin 2015; Ishida et al. 2016). In addition, *in vivo* tractography studies performed in humans have demonstrated that the cerebral peduncle is an important convergence pathway of the motor efferent system (Hagmann et al. 2003; Parker and Alexander 2005). Therefore, it is conceivable that forelimb amputation disrupts efferent pathways, and since they are packed in the cerebral peduncle, the detection of the corresponding alterations by DTI would be more robust therein.

The plastic potential of the corticofugal pathway has already been shown by a previous study (Otte et al. 2015). Adult rats that underwent unilateral hemispherectomy presented an increase of white matter volume of the contralateral cerebral peduncle, as measured by DTI techniques. This volume gain can be attributed to a compensatory plasticity for the loss of the contralateral hemisphere.

Development of the rat corticospinal pathway occurs mainly after birth (Donatelle 1977; Jones et al. 1982; Joosten et al. 1992; Oudega et al. 1994). In most mammals, this pathway has ipsilateral connections during early stages of postnatal development, which are later eliminated (O’Leary et al. 1992). This loss of connections occurs by axonal elimination followed by death of a few neurons (Oudega et al. 1994). Thus, it is expected that amputation a few days after birth leads to a more robust plasticity of the corticofugal pathway than if made in adult individuals.

In sum, we have shown that, among many other effects described by different authors (see Bahia et al. 2018, for references), limb amputation in rats impacts on myelination of different brain traits such as the corpus callosum and the cerebral peduncle. Additionally, the impact on myelination of callosal fibers fits the classical expansion of limb representation within topographic maps in S1, considered to be a result both of lower levels of excitation coming from the (absent) periphery, and of lower levels of inhibition provided by the undermyelinated corpus callosum. In addition, map expansion accords with single-axon terminal arbors expansion, as detected in another set of experiments by our group (Bahia et al. 2018). Physiological experiments are required to verify whether these inferences taken from structural analyses are confirmed directly.

## Notes

F.T.M. and R.L. designed research; R.J.V-B, C.P.B., and A.S. performed research; F.T.M., R.L., R.J.V-B., and K.M. analyzed data; and R.J.V-B., F.T.M., and R.L. wrote the paper. We thank Camila Gomes and Camila Lopes for technical assistance on the immunohistochemical techniques, Rachel Rachid for the great help on electron microscopy techniques, and Fernanda Meireles for assistance on MRI. *Conflict of interest:* None declared.

## Funding

Conselho Nacional de Desenvolvimento Científico e Tecnológico (CNPq, Grant # 465346/2014-6, INCT Program to R.L.); Fundação de Amparo à Pesquisa do Rio de Janeiro (FAPERJ, Grants # E-26/210.812/2014, E-26/202.803/2015 to F.T.M., in addition to intramural grants from D’Or Institute for Research and Education (IDOR)); PhD fellowship from CNPq (to R.J.V-B); C.P.B received a postdoctoral fellowship from CNPq during the work.
